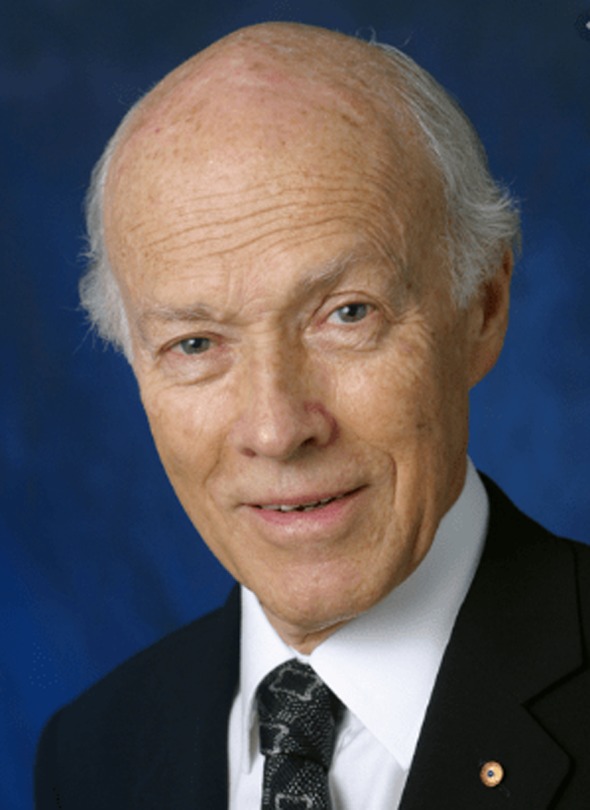# Vale Ron Penny AO (1936–2019): pioneer of clinical immunology

**DOI:** 10.1002/cti2.1119

**Published:** 2020-03-13

**Authors:** Samuel Breit, John Ziegler, Anthony D Kelleher

**Affiliations:** ^1^ St Vincent's Centre for Applied Medical Research (AMR) St Vincent's Hospital Darlinghurst NSW Australia; ^2^ Department of Immunology and Infectious Diseases School of Women's & Children's Health Sydney Children's Hospital Randwick University of New South Wales Sydney NSW Australia; ^3^ Kirby Institute University of New South Wales Sydney NSW Australia

Researchers, physicians, patients, family and friends mourn the recent loss of Professor Ronald Penny AO. He passed away on December 21 at age of 82 after a long illness.

Ron Penny was born in 1936 in Poland, but the family then moved to Australia shortly before the chaos of WWII. Ron was very proud of both his Australian citizenship and Jewish heritage and spoke of his family's migration to Australia as his second birthday. Ron was deeply devoted to his family and strongly supported by his lifetime partner, Naomi.

Taking his medical degree at the University of Sydney, Ron Penny then trained as a haematologist. Through an interest in B‐cell malignancies and their associated paraproteins, he became interested in what is now called clinical immunology very early in his career, following a study period in New York, under the tutelage Elliott Osserman, and in London with David Galton. He returned to Sydney in the late 1960s to establish the first Clinical Immunopathology Laboratory in Australia at Royal Prince Alfred Hospital. He then moved with his laboratory to St Vincent's Hospital in Sydney as a clinical academic at the University of New South Wales (UNSW). From this position, Ron Penny was a key driver in establishing the evolving fields of clinical immunology and immunopathology. His superb clinical skills, outstanding intellect, vision, ambition to make a difference and drive combined with personal charm and charisma attracted many clinicians and clinician researchers to the field, and his department grew rapidly, flourished and ultimately evolved into a prominent research centre at UNSW: The Centre for Immunology. Ron was awarded one of the first DScs at UNSW. Many senior clinical appointments in Sydney and beyond are filled by people whom he trained as clinicians, immunopathologists and researchers, most prominently the late Professor David Cooper, the founding Director of the Kirby Institute at UNSW.

Ron Penny was first and foremost an outstanding clinician with superb diagnostic skills both within and outside his speciality of clinical immunology. He could successfully devise unorthodox treatments for apparently untreatable conditions, and this, alongside his empathy and understanding for his patients' plights, led to a large clinical practice with many patients that he continued to treat over years and decades.

Ron was a polymath with wide‐ranging cultural interests and familiarity embracing classical music, visual arts literature and travel alongside his wide medical and scientific knowledge.

Ron Penny with his colleagues at St Vincent's Hospital famously diagnosed the first patient with AIDS in Australia in 1982. Very early in the evolution of this disease, Ron recognised its enormous public health implications and was a key member of a very small group of people, which guided the highly successful government response to this epidemic in Australia. Ron's intellect and personal qualities meant that he talked regularly to people at the highest levels of NSW and Australia Federal Governments and in this way was able to both formally and informally influence government policy to both health and medical research. Ron was made an Officer of the Order of Australia (AO) in 1993 for service to medical research and education, particularly in the field of clinical immunology. His passion for finding an effective evidence‐based response to AIDS was driven by his strong sense of social justice, which was deeply disturbed by this disease, and its political and social consequences, which led to stigmatisation and persecution of minority groups. This passion had its roots in his own family's experiences.

In the early 2000s, Ron left St Vincent's Public Hospital and changed his position at the University of NSW to that of Emeritus Professor of Medicine. He directed his attention to government roles including that of Senior Clinical Advisor for NSW Health and Chairman of the Corrections Health Services Board. He served as Co‐Chair of the NSW State Government's Chronic and Complex Care Implementation Group and was Member of the NSW Expert Advisory Group on Drugs, the Ministerial Advisory Council on Medical and Health Research, and NSW General Practice Council. Ron also continued in active clinical practice for over another decade until declining health forced his retirement.

Ron played a critical role in establishing the specialities of clinical immunology and immunopathology and in the establishment of a clinical research culture on the St Vincent's campus and the training and mentoring of generations of clinicians and clinician researchers. He is survived by his wife, Naomi, and his son, Mark, a renal physician, his daughter, Sheira, and five grandchildren. He will be sorely missed.

Vale Ron, clinician, teacher, researcher, mentor, friend.

Professor Ron Penny AO